# Supplementary material for: Extracellular matrix rigidity controls breast cancer metastasis via TYK2-mediated mechanotransduction
Source: Nat Commun. 2026 Mar 25;17:4392. doi: 10.1038/s41467-026-70518-9 (PMC13181009; doi:10.1038/s41467-026-70518-9)
Supplement: Supplementary file 1 — Supplementary Information [file 41467_2026_70518_MOESM1_ESM.pdf]

Extracellular matrix rigidity controls breast cancer metastasis via TYK2-mediated mechanotransduction

Zhimin Hu[1,7], Hannah E. Majeski[1,7], Aida Mestre-Farrera[1], Shirong Cai[2], Arya Lalezarzadeh[1], Yichi Zhang[1], Kei-Ichiro Arimoto[3], Dong-Er Zhang[3,4], Helen Piwnica-Worms[2], Laurent Fattet[1,6], and Jing Yang[1,5,#]

1 Department of Pharmacology, Moores Cancer Center, University of California, San Diego, La Jolla, California 92093, USA.

2 Department of Experimental Radiation Oncology, University of Texas MD Anderson Cancer Center, Houston, TX 77030, USA.

3 Department of Pathology, Moores Cancer Center, University of California, San Diego, La Jolla, California 92093-0819, USA.

4 Department of Molecular Biology, University of California, San Diego, 9500 Gilman Drive, La Jolla, California 92093-0819, USA.

5Department of Pediatrics, University of California, San Diego, 9500 Gilman Drive, La Jolla, California 92093, USA.

6Current address : Apoptosis, Cancer and Development laboratory, Centre de Recherche en Cancérologie de Lyon, INSERM U1052-CNRS UMR5286, Lyon, France.

7These authors contributed equally.

# Corresponding authors: jingyang@ucsd.edu

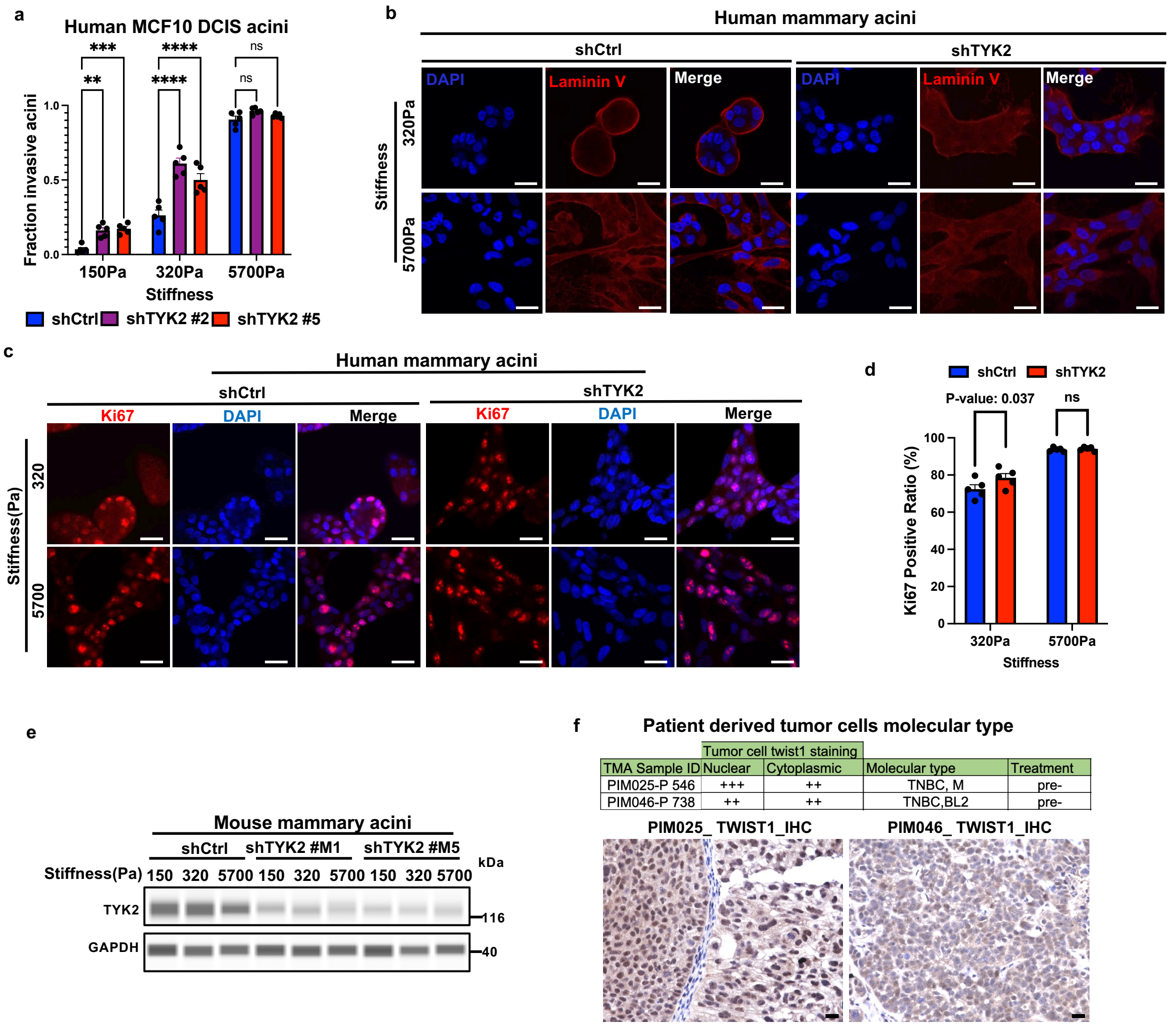

Supplementary Figure 1. TYK2 knockdown promotes EMT and invasion with minimum effect on cell proliferation under normal mammary tissue stiffness, related to Figure 1

a Quantification of invasive structures in control or TYK2-silenced MCF10DCIS acini grown on three-dimensional polyacrylamide (3D-PA) gels for 5 days, represented as the fraction of total structures(n= 5 wells per group, three independent experiments) . **b** Immunostaining of laminin V (red) and DAPI (blue) in control or TYK2-silenced human MCF10A acini grown on 3D-PA gels for 5 days, representative images from 2 independent experiments. Scale bar, 25 μm. **c** Immunostaining of Ki67 (red) and DAPI (blue) in control or TYK2-silenced human MCF10A acini grown on 3D-PA gels for 5 days, representative images from 2 independent experiments Scale bar, 25 μm. **d** Quantification of nuclear Ki67 staining in control or TYK2-silenced human MCF10A acini, represented as the percentage of total cells (n= 5 wells per group, three independent experiments) . **e** Immunoblot analysis of TYK2 expression in lysates from control or TYK2-silenced mouse Eph4Ras acini grown on 3D-PA gels for 5 days; GAPDH was used as a loading control (representative of two independent experiments). **f** Clinicopathological characteristics of human patient-derived TNBC tumors and representative immunohistochemistry staining of TWIST1 in these PDX tumors. Scale bar, 25 μm. Data are mean ± SEM, dots represent independent wells. Where indicated, quantification is shown for different wells from one representative experiment, and similar results were obtained in independent replicate experiments (see n and number of independent experiments stated for each panel), ****p < 0.0001; ***p < 0.001; **p < 0.01; ns, not significant. Two-group comparisons used unpaired two-tailed Student's t-test; multiple comparisons used one-way ANOVA with Dunnett's multiple-comparisons test. Exact P values and source data are provided in the Source Data file.

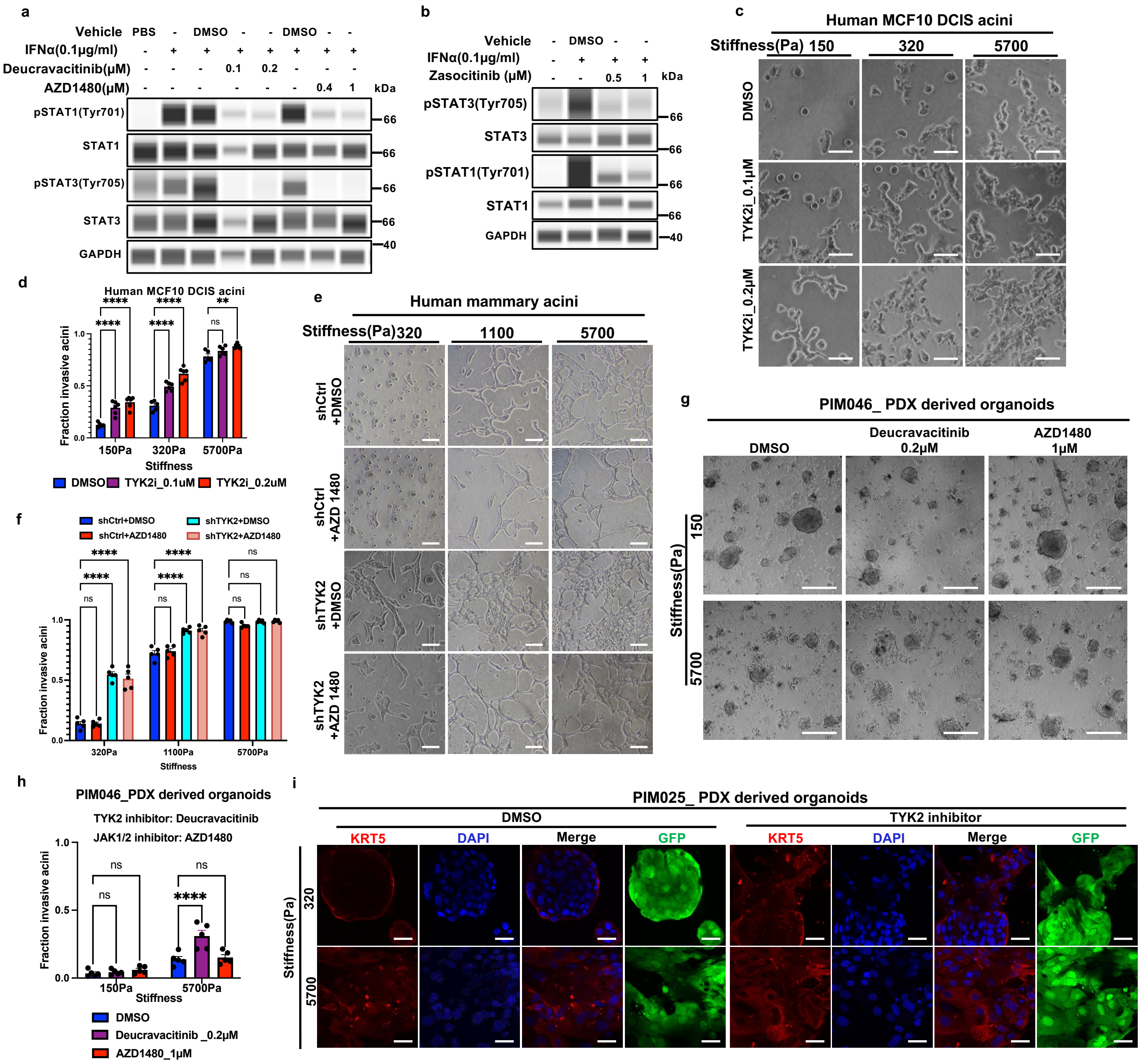

Supplementary Figure 2. TYK2 knockdown induces EMT independent of the JAK1/2 kinases, related to Figure 2

a, **b** Immunoblot analysis of lysates from human MCF10A cells treated with the TYK2 inhibitor deucravacitinib, the JAK1/2 inhibitor AZD1480, or vehicle (DMSO) for 5 days (**a**), or treated with the TYK2 inhibitor zasocitinib or vehicle (DMSO) for 5 days (**b**) (representative of two independent experiments). **c**, **d** Human MCF10DCIS acini grown on three-dimensional polyacrylamide (3D-PA) gels were treated for 5 days with the TYK2 inhibitor deucravacitinib or vehicle (DMSO) at the indicated concentrations. Representative bright-field images (**c**) and quantification of invasive structures (fraction of total) (**d**) (n= 5 wells per group, three independent experiments). **e**, **f** Control or TYK2-silenced human MCF10A acini grown on 3D-PA gels were treated for 5 days with the JAK1/2 inhibitor AZD1480 (1 μM) or vehicle (DMSO). Representative bright-field images (**e**) and quantification of invasive structures (fraction of total) (**f**) (n= 5 wells per group, three independent experiments). **g**, **h** PDX-derived organoids (PIM046) grown on 3D-PA gels were treated for 5 days with deucravacitinib, AZD1480, or vehicle (DMSO) at the indicated concentrations. Representative bright-field images (**g**) and quantification of invasive structures (fraction of total) (**h**) (n= 5 wells per group, three independent experiments). **i** PDX-derived organoids (PIM025) grown on 3D-PA gels were treated with vehicle (DMSO) or zasocitinib (1 μM) for 5 days and immunostained for KRT5 (red) and DAPI (blue); tumor cells express GFP, representative images from 2 independent experiments (**i**) (scale bar, 25 μm). Data are mean ± SEM, dots represent independent wells. Where indicated, quantification is shown for different wells from one representative experiment, and similar results were obtained in independent replicate experiments (see n and number of independent experiments stated for each panel), ****$p < 0.0001$; **$p < 0.01$; ns, not significant. Statistical significance was assessed using one-way ANOVA with Dunnett's multiple-comparisons test, as appropriate. Exact P values and source data are provided in the Source Data file.

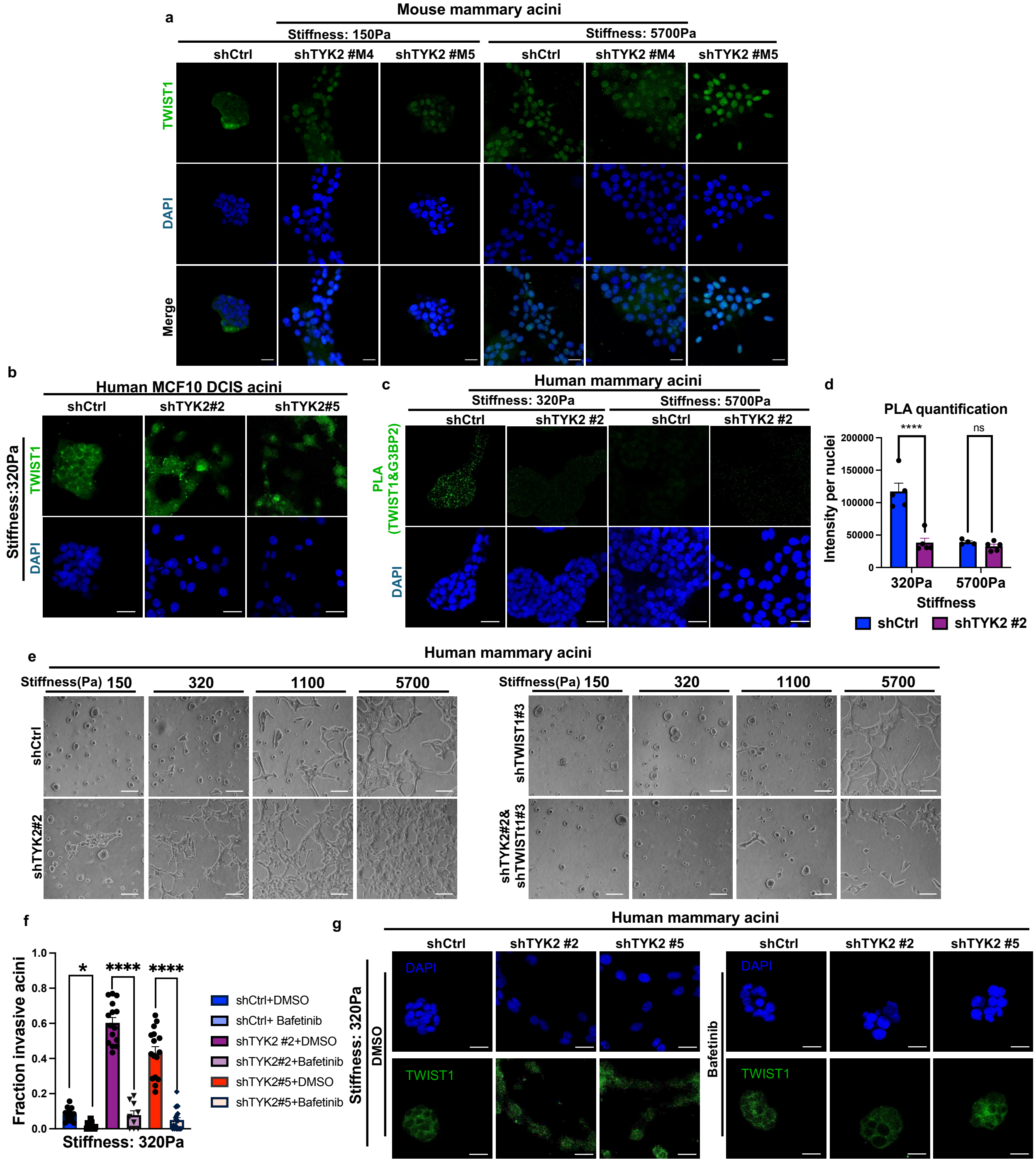

Supplementary Figure 3. TYK2 is required for maintaining TWIST1 cytoplasmic localization to prevent EMT and invasion, related to Figure 3

a Control or TYK2-silenced mouse Eph4Ras acini were grown on three-dimensional polyacrylamide (3D-PA) gels for 5 days and immunostained for TWIST1 (green) and DAPI (blue), representative images from 3 independent experiments. Scale bar, 25 μm. **b** Control or TYK2-silenced human MCF10DCIS acini were grown on 3D-PA gels for 5 days and immunostained for TWIST1 (green) and DAPI (blue), representative images from 2 independent experiments . Scale bar, 25 μm. **c** Control or TYK2-silenced human MCF10A acini were grown on 3D-PA gels for 5 days and subjected to a proximity ligation assay (PLA) to detect endogenous TWIST1–G3BP2 interactions (green dots); nuclei were counterstained with DAPI (blue). Scale bar, 25 μm. **d** Quantification of PLA signal intensity per cell; each dot represents one biologically independent sample (n = 4). **e** Bright-field images of control, TYK2-silenced, TWIST1-silenced, and TYK2/TWIST1 double-silenced MCF10A acini grown on 3D-PA gels for 5 days. Scale bar, 100 μm. **f** Quantification of invasive structures in control or TYK2-silenced MCF10A acini grown on 3D-PA gels for 5 days in the presence or absence of the LYN inhibitor bafetinib, represented as the fraction of total structures; n= 9 wells per group, three independent experiments . **g** Immunostaining of TWIST1 (green) and DAPI (blue) in control or TYK2-silenced MCF10A acini grown on 3D-PA gels for 5 days with or without bafetinib treatment, representative images from 2 independent experiments. Scale bar, 25 μm. Data are presented as mean ± SEM. dots represent independent wells. Where indicated, quantification is shown for different wells from one representative experiment, and similar results were obtained in independent replicate experiments (see n and number of independent experiments stated for each panel), Statistical significance was assessed using one-way ANOVA with Dunnett's multiple-comparisons test ($*p < 0.05$; $****p < 0.0001$; ns, not significant). Exact *P* values and source data are provided in the Source Data file.

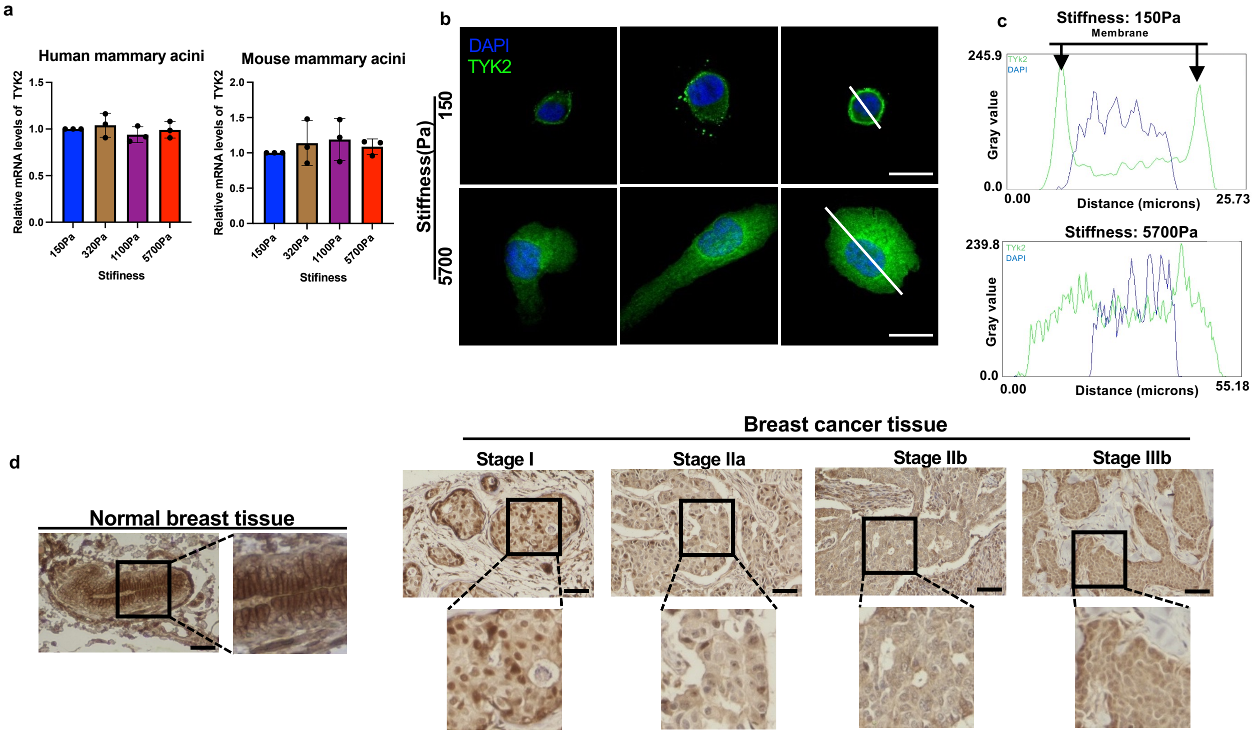

Supplementary Figure 4. TYK2 localization changes from membranous in normal breast tissues to cytoplasmic in invasive cancerous tissues , related to Figure 4

a qPCR analysis of TYK2 mRNA expression in human MCF10A acini and mouse Eph4Ras acini grown on three-dimensional polyacrylamide (3D-PA) gels, n = 3 biological replicates . **b** Immunostaining of TYK2 (green) and DAPI (blue) in human MCF10A acini grown on 3D-PA gels, representative images from 2 independent experiments. Scale bar, 25 µm. **c** Line-scan analysis showing DAPI and TYK2 intensity profiles plotted as a function of distance across acinar structures. **d** Representative immunohistochemistry images of TYK2 in a human breast tissue microarray containing normal mammary tissues and invasive breast tumors of different pathological stages, as indicated. Scale bar, 100 µm. Data are presented as mean ± SD. Statistical significance was assessed using unpaired two-tailed Student's *t*-test (ns, not significant). Exact *P* values and source data are provided in the Source Data file.

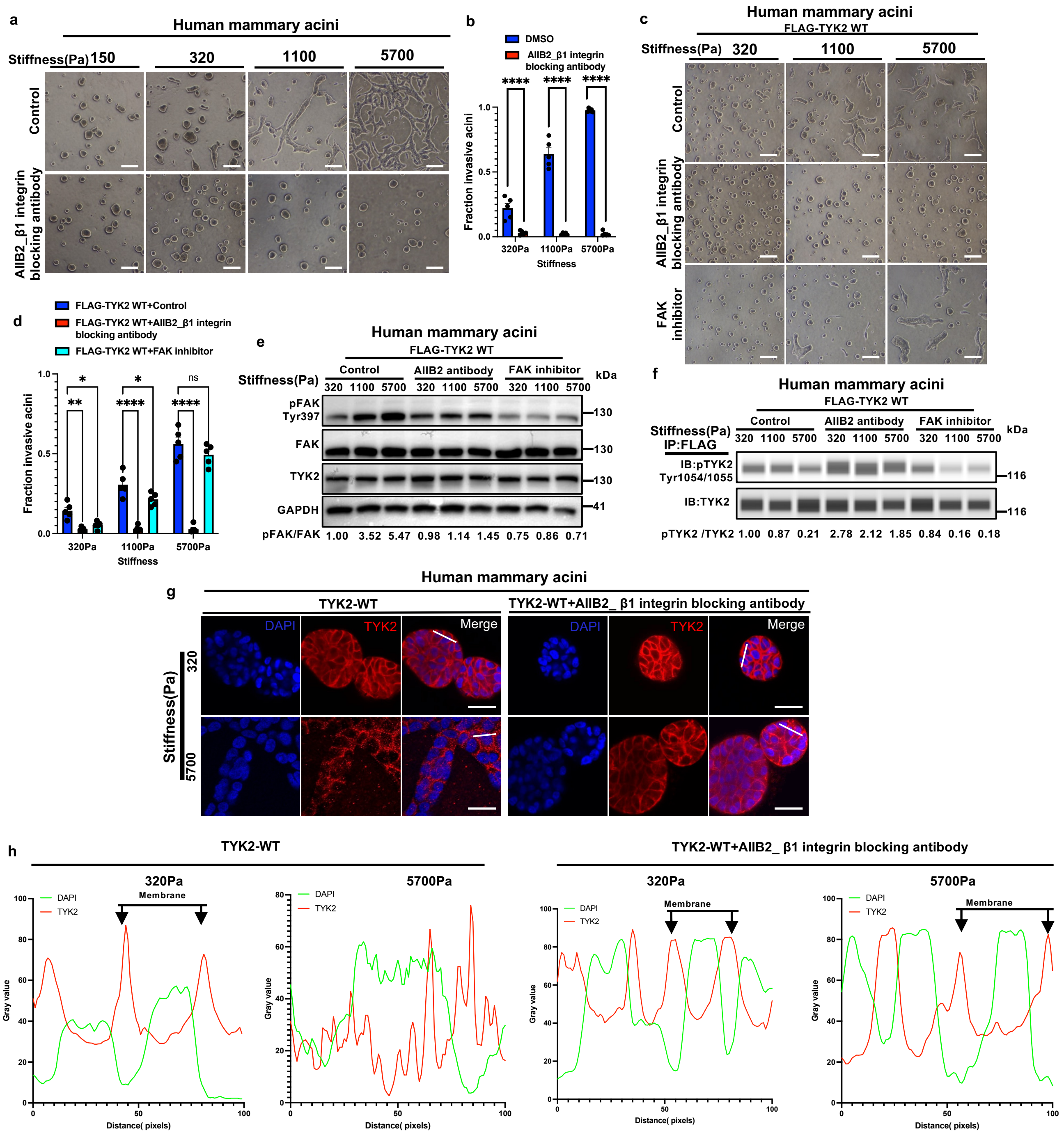

Supplementary Figure 5. The impact of β1-integrin and FAK blockade on TYK2 kinase activity and subcellular localization at various ECM rigidities.
a, **b** Human MCF10A acini grown on three-dimensional polyacrylamide (3D-PA) gels were treated for 5 days with the β1-integrin blocking antibody AIIB2 or vehicle control at the indicated concentrations. Representative bright-field images (**a**) and quantification of invasive structures (fraction of total) (**b**) (n= 5 wells per group, three independent experiments). Scale bar, 100 μm. **c**, **d** TYK2-overexpressing human MCF10A acini grown on 3D-PA gels were treated for 5 days with AIIB2, the FAK inhibitor VS-4718 (0.2 μM), or vehicle (DMSO). Representative bright-field images (**c**) and quantification of invasive structures (fraction of total) (**d**) (n= 5 wells per group, three independent experiments). Scale bar, 100 μm. **e** Immunoblot analysis of phosphorylated FAK, total FAK, and TYK2 in lysates from TYK2-overexpressing MCF10A acini treated with AIIB2, VS-4718, or DMSO and grown on 3D-PA gels; GAPDH was used as a loading control (representative of two independent experiments). **f** MCF10A acini overexpressing FLAG-tagged wild-type TYK2 were treated with AIIB2, VS-4718, or DMSO and grown on 3D-PA gels; lysates were subjected to anti-FLAG immunoprecipitation followed by immunoblot analysis as indicated (representative of two independent experiments). **g**, **h** Immunostaining of TYK2 (red) and DAPI (blue) in FLAG–TYK2–overexpressing MCF10A acini treated with AIIB2 or vehicle control and grown on 3D-PA gels for 5 days, representative images from 3 independent experiments, scale bar, 25 μm (**g**) and line-scan analysis showing DAPI and TYK2 intensity profiles plotted as a function of distance across acinar structures (**h**). Data are mean ± SEM, dots represent independent wells. Where indicated, quantification is shown for different wells from one representative experiment, and similar results were obtained in independent replicate experiments (see n and number of independent experiments stated for each panel), ****$p < 0.0001$; **$p < 0.01$; *$p < 0.05$; ns, not significant. Two-group comparisons used unpaired two-tailed Student's t-test; multiple comparisons used one-way ANOVA with Dunnett's multiple-comparisons test. Exact P values and source data are provided in the Source Data file.

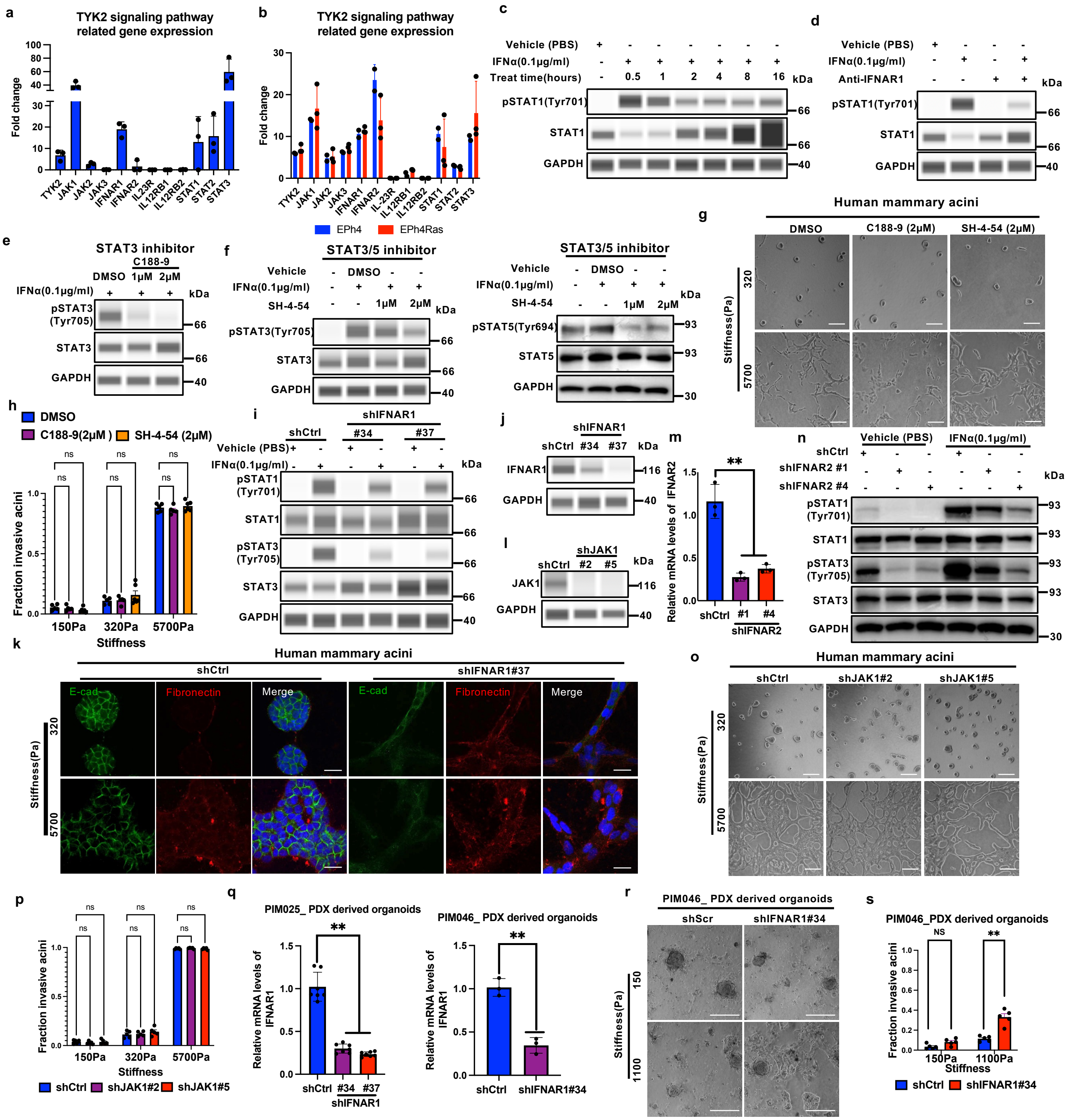

Supplementary Figure 6. TYK2 suppresses EMT and invasion at low ECM stiffness independent of the IFN/JAK/STAT signaling, related to Figure 5

a, **b** RNA-seq of JAK family members and TYK2 pathway–related genes in human MCF10A cells (GSE71862) (**a**) and mouse Eph4/Eph4Ras cells (GSE69387) (**b**). **c–f** Immunoblots of phosphorylated and total STAT1 (**c**, **d**) and STAT3 (**e**, **f**) in MCF10A cells: IFNα time course (**c**), ±IFNAR1-blocking antibody (**d**), and ±C188-9 (**e**) or SH-4-54 (**f**) under IFNα stimulation. **g**, **h** Human MCF10A acini grown on three-dimensional polyacrylamide (3D-PA) gels were treated for 5 days with C188-9, SH-4-54, or vehicle (DMSO). Representative bright-field images (**g**) and quantification of invasive structures (fraction of total) (**h**) (n= 5 wells per group, three independent experiments). Scale bar, 100 μm. **i**, **j** Immunoblots of phosphorylated STAT1/STAT1 and phosphorylated STAT3/STAT3 in MCF10A cells expressing control or IFNAR1-targeting shRNAs ± IFNα (**i**), and IFNAR1 expression in control and IFNAR1-silenced MCF10A cells (**j**). **k** Immunostaining of E-cadherin (green), fibronectin (red), and DAPI (blue) in control or IFNAR1-silenced MCF10A acini grown on 3D-PA gels for 5 days, representative images from 3 independent experiments (**k**). Scale bar, 25 μm. **l–n** Immunoblot of JAK1 in MCF10A cells expressing control or JAK1-targeting shRNAs (**l**); qPCR analysis of IFNAR2 mRNA expression (n = 3 biological replicates) (**m**); immunoblots of phosphorylated STAT1/STAT1 and phosphorylated STAT3/STAT3 in MCF10A cells expressing control or IFNAR2-targeting shRNAs ± IFNα (**n**). **o**, **p** Control or JAK1-silenced MCF10A acini grown on 3D-PA gels for 5 days: bright-field (**o**) and invasive fraction (**p**) (n= 5 wells per group, three independent experiments). Scale bar, 100 μm. **q–s** qPCR analysis of IFNAR1 mRNA expression in control or IFNAR1-silenced PDX-derived organoids (PIM025 and PIM046) (n = 3 biological replicates) (**q**). Control or IFNAR1-silenced PIM046 organoids grown on 3D-PA gels for 5 days: bright-field (**r**) and invasive fraction (**s**) (n= 5 wells per group, three independent experiments). Scale bar, 100 μm. GAPDH was used as a loading control for all immunoblot analyses; immunoblots are representative of two independent experiments. Data are mean ± SEM, dots represent independent wells. Where indicated, quantification is shown for different wells from one representative experiment, and similar results were obtained in independent replicate experiments (see n and number of independent experiments stated for each panel), ****p < 0.0001; **p < 0.01; *p < 0.05; ns, not significant. Two-group comparisons used unpaired two-tailed Student's t-test; multiple comparisons used one-way ANOVA with Dunnett's multiple-comparisons test. Exact P values and source data are provided in the Source Data file.

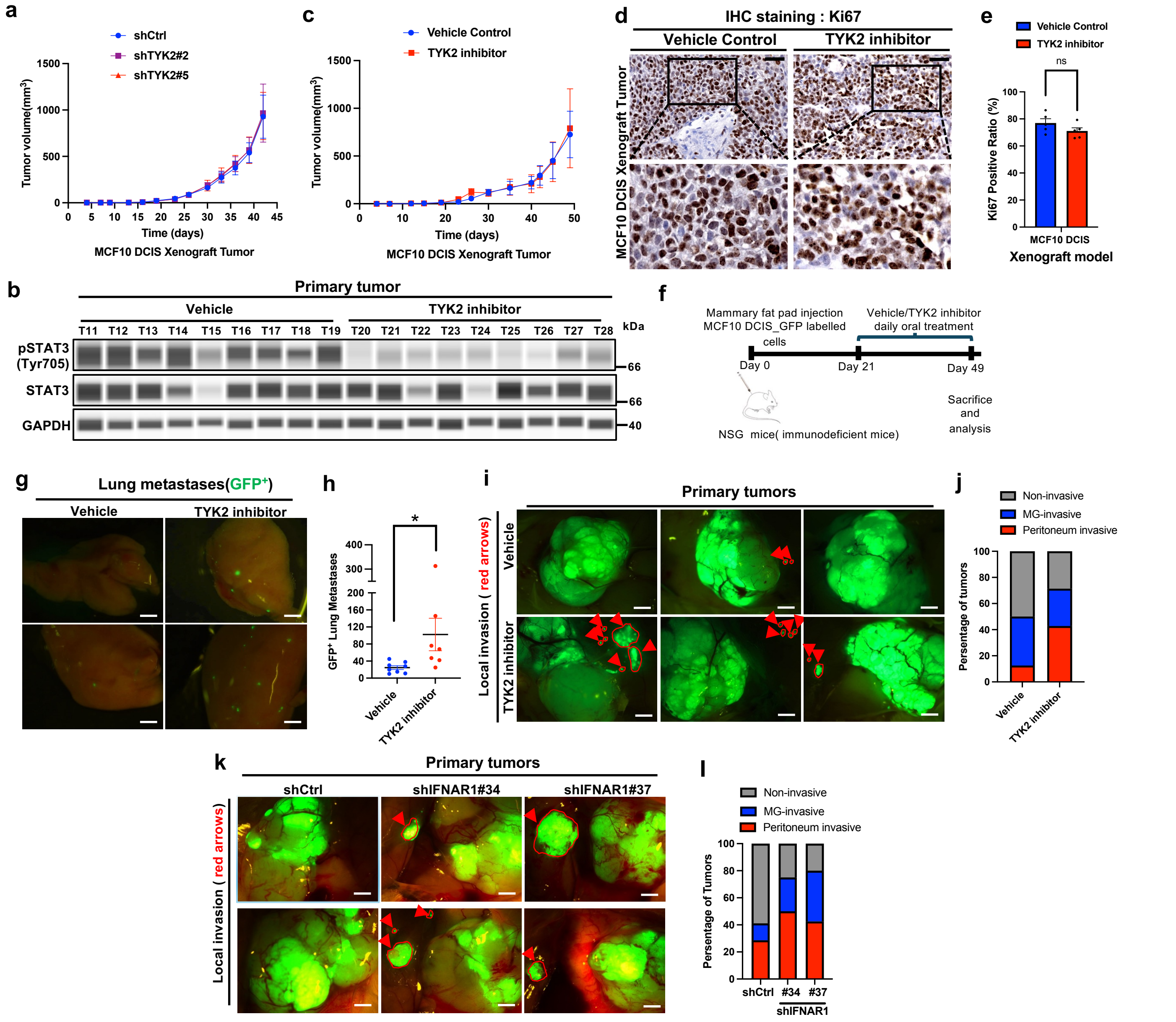

Supplementary Figure 7. Pharmacologic inhibition of TYK2 promotes breast cancer invasion and metastasis, related to Figure 6

a Tumor growth curves of MCF10DCIS xenografts expressing control or TYK2-targeting shRNAs; curves show mean tumor volume per group (n = 10 tumors per group). No significant difference was observed. **b** Immunoblot analysis of phosphorylated STAT3 and total STAT3 in primary tumors from vehicle- or deucravacitinib-treated mice; GAPDH was used as a loading control (representative of two independent experiments). **c** Tumor growth curves of MCF10DCIS xenograft primary tumors from vehicle- and deucravacitinib-treated mice (5 weeks); curves show mean tumor volume per group (n = 13 tumors per group). No significant difference was observed. **d**, **e** Immunohistochemical analysis of Ki67 (**d**) and quantification of Ki67-positive cells as the percentage of total cells (**e**) in primary tumors from vehicle- and deucravacitinib-treated mice (5 weeks) (n = 5 mice per group). Scale bar, 50 µm. **f** Schematic of the TYK2 inhibitor treatment: one million GFP-labeled MCF10DCIS cells were injected into the mammary fat pad of female NSG mice, randomized after three weeks, and treated by daily oral gavage with vehicle or deucravacitinib for four weeks. **g**, **h** Lung metastases from GFP-labeled tumors treated with vehicle or deucravacitinib: representative images (**g**) and GFP-positive nodule counts (**h**) (n = 7 mice per group). Scale bar, 1 mm. **i–l** Primary tumor invasion: representative images (arrows) and invasion incidence in vehicle or deucravacitinib-treated mice (**i**, **j** (n = 7 mice per group)) and in tumors expressing control or IFNAR1-targeting shRNAs (**k**, **l** (n = 6 mice per group).). Scale bar, 2 mm.Data are mean ± SEM, *$p < 0.05$; ns, not significant. Statistical significance was assessed using unpaired two-tailed Student's t-test. Exact P values and source data are provided in the Source Data file.

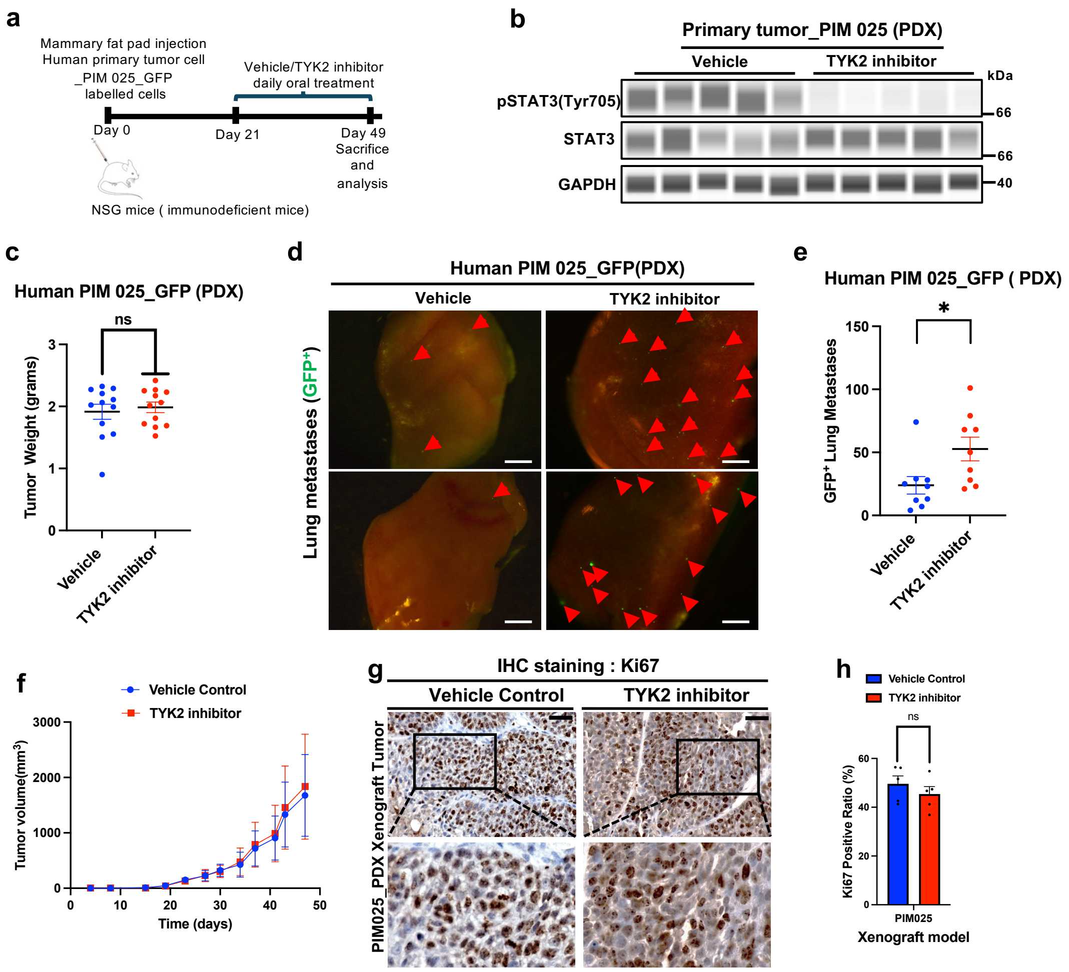

Supplementary Figure 8. TYK2 blockade promotes metastasis without impacting primary tumor growth in patient-derived breast tumor xenografts，related to Figure 7

a Schematic of the TYK2 inhibitor treatment protocol: one million GFP-labeled patient-derived xenograft (PIM025) tumor cells were orthotopically injected into the mammary fat pad of female NSG mice; after three weeks, mice were randomized and treated daily with vehicle or deucravacitinib by oral gavage for four weeks prior to endpoint analyses. **b** Immunoblot analysis of phosphorylated STAT3 and total STAT3 in lysates from PIM025 primary tumors treated with vehicle or deucravacitinib for five weeks; GAPDH was used as a loading control (representative of two independent experiments). **c** Tumor weight of PIM025 xenograft tumors treated with vehicle or deucravacitinib for four weeks at the experimental endpoint (n = 12 tumors per group). **d**, **e** Representative images of lung metastases from GFP-labeled PIM025 xenografts treated with vehicle or deucravacitinib for four weeks, with GFP-positive metastatic tumor cells shown in green, scale bar, 1 mm (**d**), and lung metastatic burden quantified as the number of GFP-positive nodules (**e**) (n = 9 mice per group). **f** Tumor growth curves of PIM025 xenograft primary tumors from vehicle- and deucravacitinib-treated groups (five weeks); curves show mean tumor volume per group (n = 16 tumors per group), with no significant difference observed. **g**, **h** Immunohistochemical analysis of Ki67 in PIM025 xenograft primary tumors, scale bar, 50 µm (**g**) and quantification of Ki67-positive cells as the percentage of total cells (**h**) (n = 5 mice per group), with no significant difference observed. Data are mean ± SEM, *p < 0.05; ns, not significant Statistical significance was assessed using unpaired two-tailed Student’s t-test. Exact P values and source data are provided in the Source Data file.

Supplementary Table 1: List of lentiviral target sequences

Target	TRC number	Sequence
**shControl (shRFP)**		CACCGGCAACAAGATGAAGAGCACCAACTCGAGTTGGTGCTCTTCATCTTGTTGTTTTTGAATTC
**Human**		
**shTYK2#1**	TRCN0000003120	CCGGGAGATCCACCACTTTAAGAATCTCGAGATTCTTAAAGTGGTGGATCTCTTTTT
**shTYK2#2**	TRCN0000003123	CCGGCGTGAGCCTAACCATGATCTTCTCGAGAAGATCATGGTTAGGCTCACGTTTTT
**shTYK2#3**	TRCN0000003124	CCGGCGAGCACATCATCAAGTACAACTCGAGTTGTACTTGATGATGTGCTCGTTTTT
**shTYK2#4**	TRCN0000320550	CCGGCGAGCACATCATCAAGTACAACTCGAGTTGTACTTGATGATGTGCTCGTTTTTG
**shTYK2#5**	TRCN0000320618	CCGGGAGATCCACCACTTTAAGAATCTCGAGATTCTTAAAGTGGTGGATCTCTTTTTG
**shIFNAR1#34**	TRCN0000059013	CCGGGCCAAGATTCAGGAAATTATTCTCGAGAATAATTTCCTGAATCTTGGCTTTTTG
**shIFNAR1#35**	TRCN0000059014	CCGGCCTTAGTGATTCATTCCATATCTCGAGATATGGAATGAATCACTAAGGTTTTTG
**shIFNAR1#37**	TRCN0000059016	CCGGGCTCTCCCGTTTGTCATTTATCTCGAGATAAATGACAAACGGGAGAGCTTTTTG
**shIFNAR1#38**	TRCN0000059017	CCGGGTTGACTCATTTACACCATTTCTCGAGAAATGGTGTAAATGAGTCAACTTTTTG
**shIFNAR2#1**	TRCN0000058784	CCGGCGCCTGATTACACAGATGAATCTCGAGATTCATCTGTGTAATCAGGCGTTTTTG
**shIFNAR2#2**	TRCN0000058785	CCGGCCATCTTATCATGGGAATTAACTCGAGTTAATTCCCATGATAAGATGGTTTTTG
**shIFNAR2#3**	TRCN0000058786	CCGGCCATCTATTGTTGAGGAAGAACTCGAGTTCTTCCTCAACAATAGATGGTTTTTG
**shIFNAR2#4**	TRCN0000058787	CCGGCCAGAAGATTTGAAGGTGGTTCTCGAGAACCACCTTCAAATCTTCTGGTTTTTG
**shJAK1#1**	TRCN0000121212	CCGGCTTCGGTTTAACCAAAGCAATCTCGAGATTGCTTTGGTTAAACCGAAGTTTTTG
**shJAK1#2**	TRCN0000121213	CCGGGCGATATATTCCAGAAACATTCTCGAGAATGTTTCTGGAATATATCGCTTTTTG
**shJAK1#3**	TRCN0000121214	CCGGCTGAAATCACTCACATTGTAACTCGAGTTACAATGTGAGTGATTTCAGTTTTTG
**shJAK1#4**	TRCN0000121215	CCGGGACAGTCACAAGACTTGTGAACTCGAGTTCACAAGTCTTGTGACTGTCTTTTTG
**shJAK1#5**	TRCN0000121216	CCGGCGAGATCTTAAGGAACCTCTACTCGAGTAGAGGTTCCTTAAGATCTCGTTTTTG
**Mouse**		
**shTYK2#M1**	TRCN0000236001	CCGGCCCAAGACATGAACCTCTATTCTCGAGAATAGAGGTTCATGTCTTGGGTTTTTG
**shTYK2#M3**	TRCN0000025886	CCGGCCCATCTTCATTAGCTGGGAACTCGAGTTCCCAGCTAATGAAGATGGGTTTTT
**shTYK2#M4**	TRCN0000025942	CCGGCCCTTCATCAAGCTAAGTGATCTCGAGATCACTTAGCTTGATGAAGGGTTTTT
**shTYK2#M5**	TRCN0000025964	CCGGCCACTTTAAGAATGAGAGCTTCTCGAGAAGCTCTCATTCTTAAAGTGGTTTTT
**shJAK2#1**	TRCN0000023650	CCGGCCGTGATCTTAACAGCCTGTTCTCGAGAACAGGCTGTTAAGATCACGGTTTTT
**shJAK2#2**	TRCN0000023649	CCGGCCTGGCAACAAGGAACATATTCTCGAGAATATGTTCCTTGTTGCCAGGTTTTT
**shJAK2#4**	TRCN0000278124	CCGGCCAACATTACAGAGGCATAATCTCGAGATTATGCCTCTGTAATGTTGGTTTTTG
**shJAK2#5**	TRCN0000023651	CCGGCGGCCCAATATCAATGGATTTCTCGAGAAATCCATTGATATTGGGCCGTTTTT

Supplementary Table 2: List of primers used for qRT-PCR

Target	Forward	Reverse
**Human**		
**TYK2**	CCAGGAGAAACCTCCAATCT	CTGGGTGATCTCCTTCTGGT
**IFNAR1**	AACAGGAGCGATGAGTCTGTC	TGCGAAATGGTGTAAATGAGTCA
**IFNAR2**	TCATGGTGTATATCAGCCTCGT	AGTTGGTACAATGGAGTGGTTTT
**JAK1**	GGAGCAGGTGGCTGTTAAAT	CCATTTCCTCCGTCTTCTGT
**JAK2**	CAGATGTTTGGAGCTTTGGA	TGTCCTTGTTTGTCATTGCC
**JAK3**	TTCGGGCTACGCAAGGATTTG	AGGCTGAGACACTCACCCT
**IL-23R**	CAGGTCACTATTCAATGGGATGC	GCAGTTCTTAATTGCTGCTTGG
**IL-12RB1**	TAGGGACCTGAGATGCTATCG	CCCGGAGCTAAGGCAACAC
**IL-12RB2**	AAAATAGATGCGTGCAAGAGAGG	GGGGAAGACCTGTGACTTGAG
**STAT1**	CTAGTGGAGTGGAAGCGGAG	CACCACAAACGAGCTCTGAA
**STAT2**	CCAGCTTTACTCGCACAGC	AGCCTTGGAATCATCACTCCC
**STAT3**	CAGCAGCTTGACACACGGTA	AAACACCAAAGTGGCATGTGA
**STAT5**	GCAGAGTCCGTGACAGAGG	CCACAGGTAGGGACAGAGTCT
**TWIST1**	AAGAGGTCGTGCCAATCAG	GGCCAGTTTGATCCCAGTAT
**GAPDH**	GAGAGACCCTCACTGCTG	GATGGTACATGACAAGGTGC
**Mouse**		
**TYK2**	CTGCCTGAGGTCACACAGAA	TAGCACCATCAAGCATCCTG
**IFNAR1**	AGCCACGGAGAGTCAATGG	GCTCTGACACGAAACTGTGTTTT
**JAK1**	CATGACTCGCTGCATGAACT	TGTTGTTGGCTGCTTTTCTG
**JAK2**	TCTGTGGGAGATCTGCAGTG	GAAAGCAGGCCTGAAATCTG
**GAPDH**	GACCCCTTCATTGACCTCAAC	CTTCTCCATGGTGGTGAAGA
