# Supplementary material for: Extracellular matrix rigidity controls breast cancer metastasis via TYK2-mediated mechanotransduction
Source: Nat Commun. 2026 Mar 25;17:4392. doi: 10.1038/s41467-026-70518-9 (PMC13181009; doi:10.1038/s41467-026-70518-9)
Supplement: Supplementary file 2 — Reporting Summary [file 41467_2026_70518_MOESM2_ESM.pdf]

nature portfolio

Corresponding author(s): Jing Yang

Last updated by author(s): 1/12/2026

Reporting Summary

Nature Portfolio wishes to improve the reproducibility of the work that we publish. This form provides structure for consistency and transparency in reporting. For further information on Nature Portfolio policies, see our Editorial Policies and the Editorial Policy Checklist.

Statistics

For all statistical analyses, confirm that the following items are present in the figure legend, table legend, main text, or Methods section.

n/a	Confirmed	
☐	☒	The exact sample size (*n*) for each experimental group/condition, given as a discrete number and unit of measurement
☐	☒	A statement on whether measurements were taken from distinct samples or whether the same sample was measured repeatedly
☐	☒	The statistical test(s) used AND whether they are one- or two-sided<br>*Only common tests should be described solely by name; describe more complex techniques in the Methods section.*
☒	☐	A description of all covariates tested
☐	☒	A description of any assumptions or corrections, such as tests of normality and adjustment for multiple comparisons
☐	☒	A full description of the statistical parameters including central tendency (e.g. means) or other basic estimates (e.g. regression coefficient) AND variation (e.g. standard deviation) or associated estimates of uncertainty (e.g. confidence intervals)
☐	☒	For null hypothesis testing, the test statistic (e.g. *F*, *t*, *r*) with confidence intervals, effect sizes, degrees of freedom and *P* value noted<br>*Give P values as exact values whenever suitable.*
☒	☐	For Bayesian analysis, information on the choice of priors and Markov chain Monte Carlo settings
☒	☐	For hierarchical and complex designs, identification of the appropriate level for tests and full reporting of outcomes
☒	☐	Estimates of effect sizes (e.g. Cohen's *d*, Pearson's *r*), indicating how they were calculated

Our web collection on statistics for biologists contains articles on many of the points above.

Software and code

Policy information about availability of computer code

Data collection	Three-dimensional (3D) immunofluorescence microscopy images were acquired using an Olympus FV1000 with 405, 488, 555, and 647 laser lines. Immunohistochemistry staining images were obtained on a Keyence BZX710 microscope. Brightfield images of acini and invasive acini in a 3D culture system were captured at 5X magnification using a ZEISS Invertoskop 40C microscope coupled with a Canon EOS 60D camera.
Data analysis	Only commercial or publicly available software was used for data analysis. 3D Immunofluorescence microscopy images were linearly analysed and pseudo-coloured using ImageJ (Fiji) analysis software. Quantification of immunoblots intensity was performed using ImageJ. Prism 10 (GraphPad, Version 10.1.1) was used for indicated statistical analysis and production of all graphs and plots.

For manuscripts utilizing custom algorithms or software that are central to the research but not yet described in published literature, software must be made available to editors and reviewers. We strongly encourage code deposition in a community repository (e.g. GitHub). See the Nature Portfolio guidelines for submitting code & software for further information.

Data

Policy information about availability of data

All manuscripts must include a data availability statement. This statement should provide the following information, where applicable:

- Accession codes, unique identifiers, or web links for publicly available datasets
- A description of any restrictions on data availability
- For clinical datasets or third party data, please ensure that the statement adheres to our policy

RNAseq data for human MCF10A cell (GEO database: accession number GSE71862) and RNAseq for mouse Eph4 and Eph4Ras cell (GEO database: accession

numberGSE69387) were obtained from publically available sources.

Research involving human participants, their data, or biological material

Policy information about studies with human participants or human data. See also policy information about sex, gender (identity/presentation), and sexual orientation and race, ethnicity and racism.

Reporting on sex and gender	N/A
Reporting on race, ethnicity, or other socially relevant groupings	N/A
Population characteristics	N/A
Recruitment	N/A
Ethics oversight	N/A

Note that full information on the approval of the study protocol must also be provided in the manuscript.

Field-specific reporting

Please select the one below that is the best fit for your research. If you are not sure, read the appropriate sections before making your selection.

- [x] Life sciences
- [ ] Behavioural & social sciences
- [ ] Ecological, evolutionary & environmental sciences

For a reference copy of the document with all sections, see nature.com/documents/nr-reporting-summary-flat.pdf

Life sciences study design

All studies must disclose on these points even when the disclosure is negative.

Sample size	For TMA analysis, sample size was predicted on sample availability and tumor stages. Tissue microarrays (TMAs) were purchased from US Biomax Inc (BR248a). A total of 24 duplicates TMA samples included 6 normal breast, 17 cases of Invasive carcinoma, and 1 case of medullary carcinoma. The TMA contained human tissues obtained with informed consent according to US federal law and are exempt from Institutional Review Board review by the University of California, San Diego Human Research Protections Program.<br>For mouse experiment, sample size was calculated by power analysis including effect size, type 1 error (p=0.05), Power (80%), two-tailed analysis, and standard deviation.
Data exclusions	No data exclusions were applied.
Replication	The experiments were repeated independently at least two times that render reproducible results.
Randomization	In all cell culture experiments, the same starting cell lines or primary cells harvested from mice were seeded in a number of plates and each plate is randomly assigned as control v.s. treatment groups.<br>For mouse experiments, all littermate mice were randomly divided into groups for implantation of different tumor cell lines .
Blinding	TMA samples were scored blindly prior to analysis.<br>Mouse tumor size, weight and lung metastasis lesions were quantified blindly.

Reporting for specific materials, systems and methods

We require information from authors about some types of materials, experimental systems and methods used in many studies. Here, indicate whether each material, system or method listed is relevant to your study. If you are not sure if a list item applies to your research, read the appropriate section before selecting a response.

Materials & experimental systems

n/a	Involved in the study	
[ ]	[x]	Antibodies
[ ]	[x]	Eukaryotic cell lines
[x]	[ ]	Palaeontology and archaeology
[ ]	[x]	Animals and other organisms
[x]	[ ]	Clinical data
[ ]	[ ]	Dual use research of concern
[x]	[ ]	Plants

Methods

n/a	Involved in the study	
[x]	[ ]	ChIP-seq
[x]	[ ]	Flow cytometry
[x]	[ ]	MRI-based neuroimaging

Antibodies

Antibodies used	TYK2 (WB 1:1000, IF 1:80, Wes 1:50, IHC 1:100) specific for human, Sigma-Aldrich HPA005157<br>TYK2 (WB 1:1000, IF 1:100, Wes 1:50,) Genetex GTX61449<br>pTyr1054/1055 TYK2(WB 1:500, Wes 1:25) Cell Signaling Technology #68790<br>TWIST1 (WB 1:200, IF 1:25, IHC 1:50) Santa Cruz Biotechnology sc-81417<br>G3BP2 (WB 1:1000, IF 1:200) Sigma-Aldrich HPA018425<br>E-cadherin (IF 1:200, Wes 1: 50) BD Biosciences #610181<br>Vimentin (IF 1:200, Wes 1: 50) Cell Signaling Technology #5741<br>Fibronectin (IF 1:200, Wes 1: 50) Sigma-Aldrich F3548<br>STAT3 (WB 1:1000, Wes 1:50) Cell Signaling Technology #9139<br>pTyr705 STAT3 (WB 1:1000, Wes 1:50) Cell Signaling Technology #9145<br>STAT1 (WB 1:1000, Wes 1:50) Cell Signaling Technology #9172<br>pTyr701 STAT1 (WB 1:1000, Wes 1:50) Cell Signaling Technology #9167<br>STAT5(WB 1:1000, Wes 1:50) Cell Signaling Technology #57580<br>pTyr694 STAT5 (WB 1:1000, Wes 1:50) Cell Signaling Technology #4322<br>EPHA2 (WB 1:500, IP, Wes 1:50) Cell Signaling Technology #6997<br>pS897 EPHA2 (WB 1:500, IP, Wes 1:50) Cell Signaling Technology #6347<br>LYN (WB 1:500, IP, Wes 1:50) Cell Signaling Technology #2796<br>pY416 SFK (WB 1:500, Wes 1:50) Cell Signaling Technology #2101<br>IFNAR1(WB 1:500, Wes 1:50) Abcam ab45172<br>GAPDH (WB 1:1000, Wes 1:5000) Genetex GTX100118<br>Flag (WB 1:1000, IF 1:500) Sigma Aldrich F3165<br>FAK (WB 1:1000,Wes 1:50) Cell Signaling Technology #13009<br>pTyr397 FAK (WB 1:1000,Wes 1:50) Invitrogen # 44-625G<br>Laminin V (IF 1:200), kind gift from M. Aumailley, University of Cologne, Germany<br>AIIb2 β1 integrin-blocking antibody(1:1000) Developmental Studies Hybridoma Bank(DSHB) #AB_528306<br>KRT5(IF 1:200) Invitrogen # PA5-32465<br>Ki-67(IF 1:200, IHC 1:200) Invitrogen # MA5-14520<br>Rabbit IgG control (IP) Cell Signaling Technology #2729<br>Goat anti-Mouse-HPR (1:10000) Jackson Immunoresearch 115-035-003<br>Goat anti-Rabbit-HPR (1:10000) Jackson Immunoresearch 111-035-144<br>Bovine anti-Goat-HPR (1:10000) Jackson Immunoresearch 805-035-180<br>Goat anti-Mouse Alexa Fluor 488 (1:200) Invitrogen A-11001<br>Goat anti-Rabbit Alexa Fluor 546 (1:200) Invitrogen A-11010<br>Goat anti-Rabbit Alexa Fluor 488 (1:200) Invitrogen A-11008<br>Goat anti-mouse Alexa Fluor 546 (1:200) Invitrogen A-11035
Validation	All antibodies used in this study were acquired from commercial sources and validated for specificity and species reactivity by manufacturer and us. Validation data is available at each manufacturer's website by searching under the provided catalog numbers.

Eukaryotic cell lines

Policy information about cell lines and Sex and Gender in Research

Cell line source(s)	MCF10A and 293T cells were purchased from the ATCC. MCF10DCIS cells were obtained from the Miller laboratory (Wayne State University, Detroit). Eph4Ras cells were obtained from the Reichmann laboratory (Zurich, Switzerland).
Authentication	All cell lines were authenticated by STR profiling.
Mycoplasma contamination	We routinely test all cell lines used using Lonza MycoAlert Mycoplasma detection kit and found them to be negative for mycoplasma consistently.
Commonly misidentified lines<br>(See ICLAC register)	No commonly misidentified cell lines were used.

Animals and other research organisms

Policy information about studies involving animals; ARRIVE guidelines recommended for reporting animal research, and Sex and Gender in Research

Laboratory animals	In this study, NOD scid gamma (NSG™) mice (IMSR_JAX:005557) were purchased from UC San Diego ACP. Mice were maintained in a specific-pathogen-free facility in ventilated cages, a maximum of 5 mice per cage, on a 12-hour day and night cycle. at 20 to 25 degrees and provided food and water ad libitum. For mammary fat pad injections, 5-8 week old mice were used. All of the mice used in the study were female.
Wild animals	The study did not involve wild animals.
Reporting on sex	Our research focuses on the breast cancer, female mice were used in this study because breast cancer predominantly occurs in

female.

Field-collected samples: The study did not involve samples collected from the field.

Ethics oversight: All animal care and experiments were performed in accordance with the animal protocol approved by the Institutional Animal Care and Use Committee of the University of California, San Diego.

Note that full information on the approval of the study protocol must also be provided in the manuscript.

Plants

Seed stocks: N/A

Novel plant genotypes: N/A

Authentication: N/A
